# Vehicle cabin air quality: influence of air recirculation on energy use, particles, and CO_2_

**DOI:** 10.1007/s11356-023-25219-x

**Published:** 2023-01-19

**Authors:** Dixin Wei, Filip Nielsen, Hannes Karlsson, Lars Ekberg, Jan-Olof Dalenbäck

**Affiliations:** 1 Division of Building Services Engineering, Department of Architecture and Civil Engineering, Chalmers University of Technology, Gothenburg, Sweden; 2 Climate Department, R&D, Volvo Car Corporation, Gothenburg, Sweden

**Keywords:** Particles, Climate, Energy, HVAC, Ventilation, Filtration, Control

## Abstract

In this study, simulations were performed to investigate the influence of different vehicle climate ventilation strategies, mainly the air recirculation (REC) degree, on the cabin air quality and climate system power. The focus of air quality is on the cabin particle concentrations including PM_2.5_ (particles of aerodynamic diameter less than 2.5 μm), UFP (ultrafine particles of aerodynamic diameter less than 100 nm), and cabin CO_2_ concentration. Three outside climates (cold, intermediate, and warm) and three outside particle concentrations are studied. The studied vehicle originally shows possibilities to meet WHO PM_2.5_ guideline of 15 μg/m^3^ with a new filter. The aged filter have reduced performance, especially when outside concentration is high. Increased REC shows advantages in all the three climates in reducing particles and climate power for the studied vehicle. Application of 70% REC (70% of ventilation air is recirculated air) on average lowers PM_2.5_ by 55% and 39% for a new and aged filter, respectively. 70% REC with a new filter reduces cabin PM_2.5_ below guideline of 15 μg/m^3^ in all conditions. The reduction of UFP counts results are generally similar to that of PM_2.5_. Increased REC also lessens the average climate system power by up to 27% on average. When REC is increased, the cabin CO_2_ concentration arises accordingly, and the magnitude is relevant to the passengers. In all studied conditions with 1 passenger, 70% REC does not increase CO_2_ above the common guideline of 1000 ppm. 70% REC is not recommended with more than 1 passengers in cold and intermediate climate and 2 passengers in warm climate. Besides, to avoid the potential windscreen fog risk in cold climate, REC should be avoided when passengers are more than 3. Except for constant REC values, a sample study investigates a dynamic control of the REC. It shows the possibility of continuously optimizing REC to reduce the climate power and particles, while maintaining the CO_2_ concentration below 1000 ppm. In warm climate with 1 passenger boarded, the average optimized REC is 90%, which in comparison with base case lead to 44% PM_2.5_ reduction and 12% climate power reduction.

## Introduction

Maintaining a good air quality level has received growing focus in the past years, for both outdoor and inside environments. One important reason is the developed awareness of potential health risk induced by the elevated particulate matter concentration. Especially the smaller particles like PM_2.5_ (particles of aerodynamic diameter less than 2.5 μm) and UFP (ultrafine particles, which have aerodynamic diameter less than 100 nm) might have higher risks of entering human respiration system and potentially human brain (Mitsakou et al. 2007; Shiraiwa et al. 2017).

Vehicle cabin is one challenging environment due to the elevated particle concentration from road environments. The HVAC (heating, ventilation, and air conditioning) system in modern vehicles is capable of treating the incoming air to desired temperature and filters part of the pollutants with the HVAC filter. The cabin particle levels are dependent on many factors. Outside particle concentration and size distribution, as well as filter efficiency, have major influence on the concentration. More particles in the transportation microenvironment and the surrounding traffic lead to more particles entering the vehicle (Kaur et al. 2005; Knibbs et al. 2009; Knibbs and de Dear 2010; Wang et al. 2015; Jain 2017). The design of the filter and the filter status affect the filter efficiency. The ventilation of the vehicle climate system also influences the concentration. Reduced airflow rate (Zhu et al. 2007; Knibbs et al. 2010; Abi-Esber and El-Fadel 2013; Jain 2017; Wei et al. 2020) and the application of recirculation are beneficial in reducing the particles (Pui et al. 2008; Qiu et al. 2017).

There are investigations to further reduce the particle levels in the cabin by better HVAC designs. Multi-layer filters and two-step filters exist in different cars. Pre-ionization of particles has been proven to improve the filtration performance in both rigs and cars (Agranovski et al. 2006; Park et al. 2011; Wei et al. 2020).

When it comes to improvement through existing climate components, recirculation (REC) of cabin air into vehicle HVAC system could be beneficial in reducing the cabin particle concentrations when the outside PM levels are higher than PM levels in the cabin, which is normally the case for modern vehicles with a relatively good HVAC filter installed (Qiu et al. 2019). This could on some occasions be energy saving if the cabin air requires less power from climate system compared with outside air. Meanwhile, recirculated cabin air could contain CO_2_ and humidity from the passengers, which are important to concern for prevention of passenger fatigue and fog risks (Mathur 2016). Mathur (2008) performed measurements in vehicles under full recirculation mode and summarized CO_2_ concentration as function of time, number of passengers, and vehicle speeds, etc. It is observed that even with only 1 passenger, the full recirculation will accumulate CO_2_ to 1100 ppm in 5 min. The application of full REC together with an air purifier in cabin was found beneficial in reducing UFPs while maintaining CO_2_ around 1200 ppm(Tartakovsky et al. 2013)_._ Partial REC was tested in a vehicle, and trade-off between nano-particle reduction and CO_2_ accumulation was investigated (Jung et al. 2017). 50–75% was found beneficial at different fan speeds.

While there is still a lack of comprehensive investigation on different REC degrees, i.e., the proportion of ventilation air coming from recirculated cabin air, from 0 to 100%, there is a demand to correlate the air quality performance together with the vehicle climate system performance, which improves the understanding of the REC influence on the complete vehicle.

This study aims to investigate different REC levels to improve the cabin air quality, as well as the corresponding impacts on climate system energy consumption. The application possibilities in climate control, the limitations from CO_2_, and humidity are investigated. The different REC levels are compared under various environmental conditions, including outside meteorologic conditions, outside pollution condition, and passenger numbers.

## Methods 

First, a brief description of the simulation model is given. Second, the details of the specific test cycles developed for this study are explained and the corresponding parameter settings for the climate system operation are described. Third, the total 67 test cases to implement different recirculation degrees are summarized. Finally, the compared results in different cases are explained.

### Background: simulation model description

This study utilizes two existing models, one vehicle climate system model (Nielsen et al. 2015) and vehicle cabin air quality model (Wei et al. 2022), where the latter model has been developed as an extension of the former model. Here, the models are described briefly. For more details, please see “Methods” of the corresponding article.

Firstly, the climate system was simulated with the software GT-SUITE, which solves Navier–Stokes equation in one dimension. The focus was on the energy use of the interior climate system. It consists of sub models of the passenger compartment, the air-handling unit, the AC (air conditioning), and a climate coolant circuit. The climate components are simulated in detail. The climate control system is also integrated to the model, so it operates as in real production vehicles. The target of automatic climate control is to provide comfort for the customers with good energy efficiency. The control considers multiple inputs including ambient temperature, humidity, sun load, etc. and gives outputs for controlling the HVAC fan, operation of the heater and cooler, etc. The control on REC also takes input including outside air quality, fogging risks etc.

**Fig. 1:**
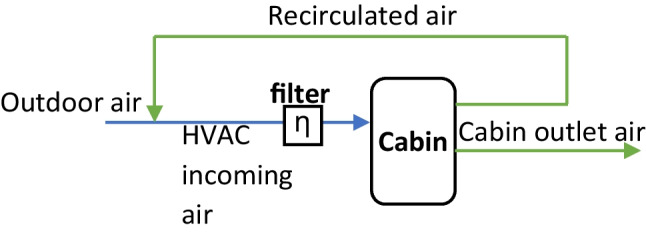
Flowchart of the HVAC incoming air and filtration in the climate system model

Secondly, the extension of air quality model is developed to simulate particle and CO_2_ concentration in the cabin. The outdoor air and recirculated air from the vehicle cabin are mixed and then filtered at the HVAC filter. The percentage of the recirculated air, i.e., REC degree, could vary between 0 and 100%. A simple flowchart is given in Fig. 1. Particles in the incoming air and size-resolved filtration of particles in the HVAC filter were simulated in detail based on available component tests. Particles from sources other than the ambient air, e.g., passengers, were omitted in this model. Besides, the internal source of CO_2_ from human respiration, deposition of particles and infiltration flows were simulated based on relevant studies. The model could be used to simulate cabin concentration of particles of various sizes between 10 nm and 2.5 μm as well as cabin CO_2_ concentration.

Both models have been validated against various road measurement data and showed good agreement (Nielsen et al. 2015; Wei et al. 2022). The mean absolute percentage error for the electrical power in the climate model was between 6 and 13%, and the mean absolute error for cabin temperature was less than 2.4 °C. The simulated particles and CO_2_ correlate well with measurement at different filter statuses and locations (Person’s *r* 0.89–0.92). With these models, the simulation in this study provides results of the energy use for the major climate components, air quality levels in the cabin, and other climate performances simultaneously. The required input to the model includes the meteorological conditions, ambient concentrations of particles and CO_2_, and the vehicular parameters (ventilation setups, vehicle speeds, HVAC filter efficiencies, etc.).

### Test cycle development

The cabin air quality level and climate energy use depend on the ambient environment and how the climate system operates accordingly. The test cycle used in the study is aiming at achieving a more comparable and representative investigation under different outside conditions. The main focus is on the variation of ambient temperature, humidity, sun load, and the ambient particle concentrations. The details are now explained.

#### Ambient environment: meteorological parameters

One important factor when comparing the vehicle climate system is to compare similar and relevant conditions. The vehicle climate system could operate in different modes depending on the outside condition. To achieve a more thorough investigation, a previously developed test cycle has been utilized to simulate three outside climates (Nielsen and Uddheim 2016). The three climates are summarized in Table 1, each weighted for occurrence. In brief, the conditions are calculated based on the ambient data in 15 largest markets for Volvo Cars, weighted with sales distribution and a common vehicle departure time distribution. In the intermediate (T15) and warm (T27) climates, the vehicle has one-hour sun soak before the cycle. The sun elevation is 37° in T15 and 50° in T27. The azimuth angle changes 5° per second, completing a revolution in 72 s. For more details, please see “Methods” of the corresponding article (Nielsen and Uddheim 2016).

**Table 1:** Ambient conditions of the three climates

	Weight	Temperature (°C)	Dewpoint (°C)	Sun load (W/m^2^)
Cold (T0) Intermediate (T15) Warm (T27)	0.2 0.6 0.2	0 15 27	− 4 7 17	0 200 400
Weighted average		14	7	200

#### Ambient environment: particle and CO_2_ concentration

The cabin particulate matter concentrations are highly influenced by the outside concentrations, i.e., the particles that are entering the vehicles, as well as the vehicle’s incoming air filtration system. Both the outside particle mass and count concentration in turn could vary significantly in different locations and road conditions. Huang et al. (2012) have reported outside PM_2.5_ concentration of 35 μg/m^3^ in Beijing, China, from a previous on-road measurement. Compared to that, Wei et al. (2020) have encountered average outside PM_2.5_ concentration of 167 μg/m^3^ while driving in several Northern China cities. The outside UFP count concentrations, in several studies, had variation between 22 × 10^3^ p/cm^3^ and 1700 × 10^3^ p/cm^3^ (Zhu et al. 2007; Knibbs et al. 2009).

To investigate the cabin air quality at different ambient conditions, three previously measured road particle concentration profiles from Sweden and Northern China have been selected as low, medium, and high outside concentrations (Wei et al. 2020). These concentrations were measured with the Grimm MiniWRAS (Mini Wide Range Aerosol Spectrometer) model 1.371, which measures particles of aerodynamic diameter from 10 nm until 35 μm, distributed into 41 size channels. Each profile represents average of at least 5 min of stable data. The PM_2.5_ and UFP concentrations of the profiles are given in Table 2. The corresponding detailed particle mass and count concentrations from 10 nm to 2.5 μm are given in Appendix Table 6.[Edit]

**Table 2:** Three levels of outside particle concentration profiles used in the simulation, as input to the model

	PM_2.5_ (μg/m^3^)	UFP counts (N/cm^3^)	EEA guideline level
Low	18	~ 7000	Fair
Medium	48	~ 20,000	Poor
High	128	~ 30,000	Extremely poor

According to guidelines on European Air quality Index from European Environmental Agency, they lie within the fair, poor, and extremely poor air quality levels correspondingly (European Environment Agency 2013). For details on the explanation of the total 6 index levels, please see the Appendix Table 7.

The initial inside particle concentration is assumed the same as outside concentration, considering the door/window opening and no pre-cleaning function is applied.

A new and a 500 h-aged (end-of-service) filter, which are the same model as in the simulated vehicle, have been tested in previous rig measurements. The size-dependent efficiencies were already utilized in the air quality model (Wei et al. 2022). The detailed values are given in Appendix Table 8. To represent the actual usage of filter in customer driving, the filter aging was performed in an HVAC rig with access to outdoor air in 2018 April at Shanghai. Ventilation fan speed was set to low (1430 rpm), no recirculation. 500 h correlates to around one-year driving, the recommended filter service interval in China.

The outside CO_2_ concentration is required as input to the model. Several studies have reported elevated on-road CO_2_ concentration compared with background concentration. Wei and Wang (2020) have reported average on-road CO_2_ concentration of 473 ± 34 ppm, in a measurement campaign performed on expressways in Shanghai. Larson et al. (2017) have concluded average CO_2_ of 557 ppm in a study route with significant truck traffic, in the city of Seattle. In another data collection on highways in Minnesota, average concentration of 404 ppm have been reported (Kittelson et al. 2004). For this study, the average of three reported values—478 ppm—is used as the estimated outside CO_2_ concentration.

#### Other vehicle settings in the test cycle

The studied vehicle is a Volvo XC90 (model-year 2018, PHEV) with estimated cabin volume of 4.1 m^3^, which utilizes a high voltage coolant heater (HVCH) and an electric compressor. The vehicle is running in electric mode that the engine is not a heat source. The vehicle velocity profile is the Worldwide Harmonized Light vehicles Test Procedure (WLTP) class 3 (UNECE 2014). The length of the cycle is the length of WLTP, i.e., 30 min. The climate system is running in automatic mode with temperature setting of 22 °C.

The in-cabin source of CO_2_ from passenger respiration is simulated. It is defined by the gas volume exhaled from a person’s lung per minute (L/min) multiplied by the CO_2_ concentration contained in the exhaled air (ppm). An average rate of 6.5 L/min is used in this study since passengers sitting in the stand-still car were almost at rest (Levitan 2015). Concentration is set to 40,000 ppm based on a relevant study (Scott et al. 2009).

In this study, some simplifications and modifications on the climate control are applied in comparison with auto mode in production vehicles, mainly for the purpose of simulation need and achieve fairer comparison in the different cases. First is the REC degree which is controlled manually to different levels (see later in “Simulated cases”). The same applies to the evaporator set point, i.e., the air temperature after evaporator, as well as whether AC is on or off. In this study, in cold (T0) and intermediate (T15) climate, AC is off. In warm climate (T27), AC is on with evaporator set point of 12 °C. While in reality, these parameters are varied depending on many climate control inputs, such as the environmental and cabin temperatures. Another example is that feedback from an ambient air quality sensor could request for temporary REC to avoid outdoor pollutants, e.g., driving in a tunnel.

Another simplification is the air distribution mode in the cabin. For T15 and T27 cases, a vent mode of air distribution is used, i.e., a major portion of the ventilation air is out from the chest level ducts. For the T0 cases, a defroster/floor air distribution mode is selected, which in the utilized model means around 60% percent of air is distributed to the defroster and small amount to the chest level. While in more realistic conditions in T0, air distribution would possibly start with the defroster/floor mode for a while and alter during the driving cycle. A more complex strategy is applied depending on the temperatures and potential windscreen fog risk, etc.

Moreover, to achieve a fairer comparison on the climate power, a similar cabin temperature profile should be reached in cold (T0), intermediate (T15), and warm (T27) climate separately. Meanwhile, for fairer comparison of particle and CO_2_ concentrations, the airflow or the fan speed should be similar between cases. The applied strategy in T0 and T15 cases is that for higher recirculation cases, the maximum allowed heater power is reduced to reach a similar heat-up speed. For T27 cases, the compressor in this study is set to reach the same air temperature after the evaporator (the evaporator set point); thus, a self-control ensures the same temperature profile.

### Simulated cases

Different REC degrees, which is the proportion of ventilation air coming from recirculation, are investigated in different outside conditions. The outside conditions vary at three climates and three particle concentrations. A variation of passenger numbers is also considered. In the end, a feedback control to continuously adjust the REC degree based on the cabin CO_2_ concentration is applied, to investigate the optimal REC in different cases. A summary of all the simulated scenarios are given in Table 3, and details are explained in each sub-section.

**Table 3:** A summary of all the simulated scenarios in the study

	Ambient conditions	Outside particle concentration	REC	Passengers	Total no. of cases
Base cases	T0, T15, T27	Low, medium, high	T0 and T15: 0% T27: 50%	1	9
Increased recirculation 1 passenger	T0, T15, T27	Low, medium, high	T0 and T15: 30%, 50%,70% T27: 70%	1	21
Increased recirculation 2–5 passengers	T0, T15, T27	Medium	T0 and T15: 30%, 50%,70% T27: 70%, 90%	2, 3, 4, 5	32
Feedback control on REC	T27	Medium	Controlled	1, 2, 3, 4, 5	5

#### Base cases

The base cases represent the current status in the vehicle. Three ambient conditions, T0, T15, and T27, as in Table 1, and three levels of outside particle concentration, low, medium, and high, as in Table 2, are simulated with one passenger. The REC degree is constant. In the T0 and T15 cases, REC is 0%, and in T27 cases, 50% REC applied. One passenger is simulated based on that the latest average occupancy rate of passenger cars in EU countries was around 1.45 passengers per vehicle (European Environment Agency, 2015).

#### Increased recirculation with 1 passenger

Compared with base cases, REC is increased while the rest remain the same. For T0 and T15, REC degrees are increased from 0 to 30%, 50%, and 70%. For T27, REC is increased from 50 to 70%. The outside particle concentration is still varied at low, medium, and high levels as in Table 2, and 1 passenger is simulated.

#### Increased recirculation with 2 to 5 passengers

In the utilized simulation model, the cabin particle concentration would not be affected by the number of passengers. While the cabin CO_2_ concentration and windscreen fog risk will be influenced. To understand the influence from increased REC on these two results, 2, 3, 4, and 5 passengers are simulated. Similarly, as before, three different outside temperatures are used. For T0 and T15, REC degrees are increased from 0 to 30%, 50%, and 70%. For T27, REC degree is increased from 50 to 70% and 90%.

#### Feedback control of recirculation

All the above cases all have constant REC in the cycle. Stepping forward, the REC can be dynamically adjusted, which is more connected with real production vehicles.

Thus, a PI control unit is applied in the simulation model to control the REC degree (0–100%) based on the cabin CO_2_ concentration, which is the same control type as the fan and heater controller (PID) used in the studied vehicle’s climate control. The control target of CO_2_ concentration is lower than 1000 ppm, which has been recommended by several indoor environmental guidelines/standards, to achieve a good indoor air quality (Lowther et al. 2021). The utilized proportional gain is − 0.03 and integral gain is − 0.0003 of the PI unit. In this study, a sample simulation in warm climate (T27) with feedback control is displayed. It investigates the strategy of continuously optimizing the REC to reduce the energy consumption and cabin particles, as well as maintain an acceptable CO_2_ concentration, in combination with usage of sensors in the vehicle.

### Compared results

Table 4 contains an explanation about the results that are compared. Regarding the power consumption of the main climate components, the heater, compressor, and fan are included (Nielsen et al. 2016). The vehicle cabin is divided into 28 air volumes in the model. The cabin CO_2_ concentration and particle concentrations are volume weighted average of the whole cabin.

**Table 4:** Compared results

Parameter	Compared value	Notes
Cabin PM_2.5_ concentration	Steady state of volume averaged PM_2.5_ concentration	The particle concentrations normally settle down to steady values within first 5 min. Thus, the steady-state results are compared
Cabin UFP counts concentration	Steady state of volume averaged UFP concentration	Same as above
Climate power	Case average (30 min average)	
Cabin CO_2_ concentration	Transient profile of volume averaged CO_2_ concentration	
	Case average (30 min average)	Only in the feedback control simulation
Windscreen fog risk	Time with potential risk	Potential risk when windscreen temperature and cabin air dew *T* difference less than 5 °C

Steady-state particle concentrations are compared since the inside particles normally settle down rapidly. Climate power however is more diverse in the whole cycle, so the 30-min average is compared. Transient profile of the CO_2_ concentration is monitored as the cycle average may not reflect high exposure in part of the cycle.

## Results

In this section, all the results from cases in Table 3 are presented, in the sequence of base cases, increased recirculation cases with different passenger numbers, and sample simulation for feedback control on recirculation.

### Base cases

As described in Table 4, the particle concentration of PM_2.5_, UFP counts are presented as steady-state values. The climate main power consumptions are cycle average values (30 min). CO_2_ concentration is predicted as transient profiles.

#### Steady-state results of inside PM_2.5_ and UFP

**Fig. 2:**
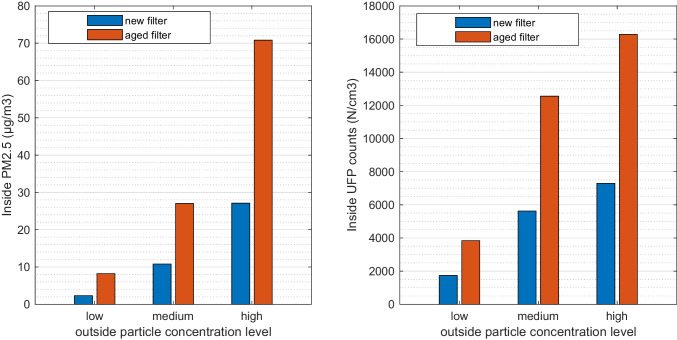
Steady state inside PM_2.5_ concentration and UFP counts at three outside concentration levels, grouped with new and aged filter. Low, medium, and high stand for outside PM_2.5_ concentration, which are 18, 48, and 128 μg/m^3^ correspondingly. Each bar is the weighted average of T0, T15, and T27. One passenger is simulated

Steady-state results of PM_2.5_ and UFP counts are compared in Fig. 2. The results are grouped by new and aged filters, categorized in three outside particle concentration levels. Each bar in the figure is the weighted average result of the three ambient conditions (T0, T15, and T27). The particle concentrations from three climates are in fact similar due to the only difference is a slight variation on the ventilation airflows.

The latest WHO guideline indicates the 24-h average of PM_2.5_ concentration to be below 15 μg/m^3^ (WHO 2021). It could be seen that with the new filter it is able to meet guideline levels in the cabin at low and medium outside particle levels. While if an end-of-service filter is still in use, in medium and high outside pollution concentrations, the cabin air quality does not meet the guideline. The similar trend can be seen for UFP counts. A slight difference is that at medium outside particle level, inside UFP counts with the aged filter is obviously higher than that at high outside particle level with a new filter, which is not the case for PM_2.5_.

The in-cabin particles are highly influenced by outside concentrations and the filter efficiency. One direct indication from Fig. 2 is that the filter should be serviced properly to maintain a good efficiency, which requires involvement from both customers (knowledge) and manufacturers (design of service interval based on measurement/model data). The filter in this study is a multi-layer electrostatically charged synthetic filter made of polypropylene and active carbon, which is common in premium modern vehicles. Enhancing the filter efficiency, for example with HEPA (high efficiency particulate air) filters, would be an effective measure to maintain a good cabin air quality level regardless of outside conditions. While this would normally lead to an increase of pressure drop due to filter media design, which means elevated fan power, a well-balanced design is required to ensure acceptable pressure drop and space for an elevated efficiency.

#### Transient profile: cabin PM_2.5_ and CO_2_ concentration 

**Fig. 3:**
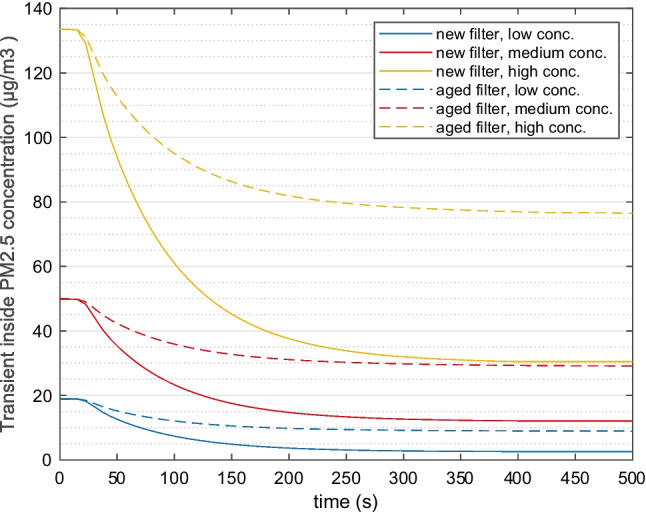
Examples of inside PM_2.5_ concentration profile in the 30-min simulation cycle. Ambient condition T0. Outside particle concentration of low, medium, and high. New and aged filters are compared. One passenger

In the start of the 30-min cycle, the cabin particle concentrations are assumed to the same as outside concentrations, when the doors are open. Subsequently with the doors closed and the ventilation starts operation, particles are captured at the filter. Thus, particle concentrations drop to steady values relatively fast, normally within 5 min. Examples of transient PM_2.5_ profile are presented in Fig. 3. The outside temperature is 0 °C. Different outside particle concentrations and filters are compared.

**Fig. 4:**
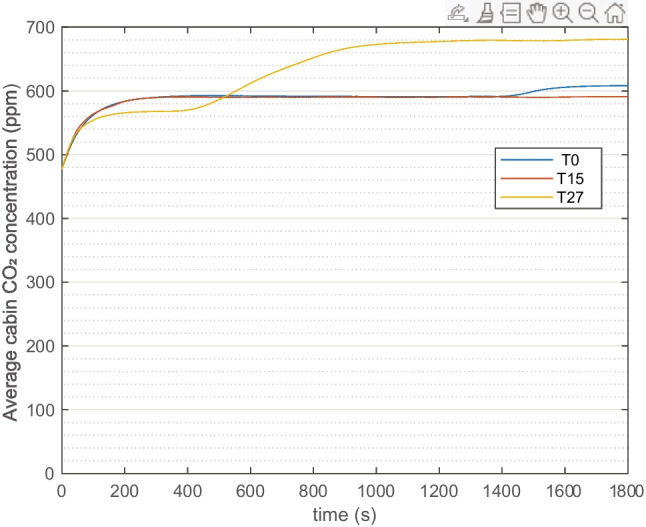
Base case cabin CO_2_ concentration profile at three ambient conditions (T0, T15, and T27), 1 passenger is simulated

The average cabin CO_2_ concentration is displayed for the whole 30-min simulation in Fig. 4. The climate control strategy applied in three climates lead to the different results. Overall, the fan rpm is adjusted by the climate control in the cycle, normally higher in the beginning due to cooling and heating demand, and lower when the cabin temperature is closer to desired value. This together with passenger respiration explain the general increase of concentration. A difference to be noted for T27 is that fan rpm is reduced later in time sense and higher in magnitude compared with T0 and T15. This leads to the more obvious increment of concentration around 600 s for T27.

The T0 and T15 results are similar because the fan operates at similar rpm and zero recirculation. T27 case has a slightly higher airflow, but with 50% REC, which results in higher CO_2_ concentration. In all three climates, the CO_2_ concentration is always lower than the 1000 ppm, mainly due to only 1 passenger being simulated.

#### Energy consumption

**Fig. 5:**
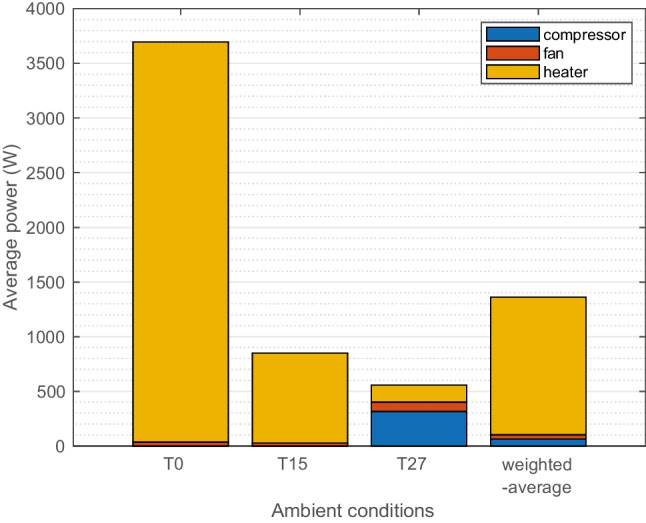
Case average power of compressor, heater, and fan at T0, T15, and T27. Weighted average (weight 0.2, 0.6, and 0.2)

The base case results of main climate component power are compared in Fig. 5. The results are 30-min average. At T0, the high-voltage coolant heater is the main consumer to reach a desirable 22 °C air temperature in the cabin, together with zero compressor power and low fan power. T15 has lower heating demand. At T27, the power consumption is relatively low and mainly is composed of the compressor. This compressor power (around 300 W) is slightly lower than the result (around 470 W) from a previous simulation for T27 using a mechanical compressor (Nielsen et al. 2016); this is mainly due to a relatively efficient electric compressor is used. The weighted average of the three climate is 1.4 kW.

It is important to repeat here the applied climate operation in this study, that the AC is off in T15 condition. It is mainly considering the dehumidification need is low in the T15 condition (see Table 1: ambient conditions of the three climates) This is a simplification compared to real control, where the AC operation could be varied frequently. The results represent the climate consumption in PHEV cars without heating from the engine.

### Increased recirculation with 1 passenger

In this section, the REC is increased to a fixed level throughout the entire cycle. For T0 and T15, recirculation is altered to 30%, 50%, and 70% and for T27 to 70%. Passenger number is still 1, same as the base case. Increased REC demonstrates the ability to reduce particle concentrations and climate power in different environments.

#### Steady-state results of inside PM_2.5_ and UFP

**Fig. 6:**
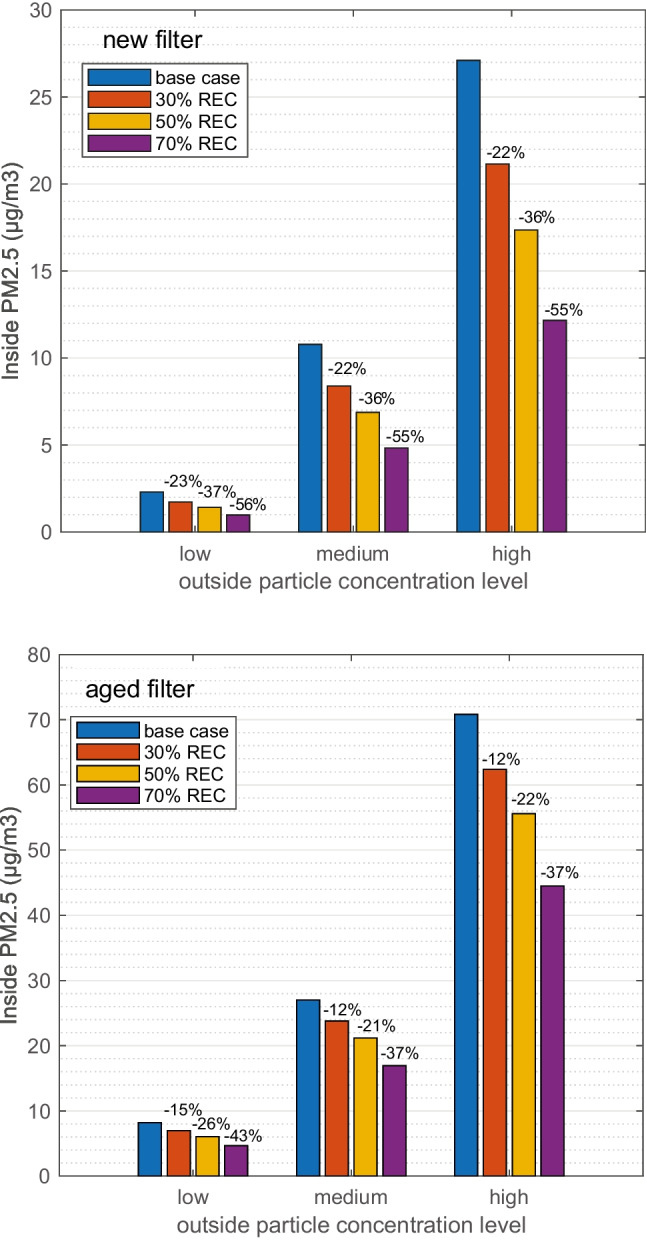
Steady state inside PM_2.5_ concentration at three outside concentration levels, grouped with different recirculation degrees, in comparison with base case. Low, medium, and high stand for outside PM_2.5_ concentration, which are 18, 48, and 128 μg/m^3^ correspondingly. Each bar is the weighted average of cold (T0), intermediate (T15), and warm (T27) climate. One passenger is simulated

In Fig. 6, the steady-state results of inside PM_2.5_ concentrations with increased REC are compared with base-case results (Fig. 2). The results are grouped by REC degrees, categorized in three outside particle concentration levels. Each bar in the figure is the weighted average result of the three climates. The results indicate that the inside particle concentrations could be reduced with increased recirculation. For example, when 70% REC is applied with a new filter installed, even at high outside particle concentration the inside PM_2.5_ is below the guideline level of 15 μg/m^3^, which means 55% percent reduction is achieved in comparison with base case (27 μg/m^3^). When it comes to aged filter, increased REC is also leading to a better cabin air quality level. At high outside concentration, 70% REC results in 37% reduction of PM_2.5_. Even with 30% REC, both new and aged filter achieved on average 22% and 13% reduction. The similar comparison on UFP counts is given in Appendix. Fig. 13

It should be noted that, for 30% REC bars, the calculation is based on the base case result of T27, which is already running at 50% REC, i.e., weighted average of T0 30% REC, T15 30% REC, and T27 50% REC.

#### Transient profile: cabin CO_2_ concentration 

**Fig. 7:**
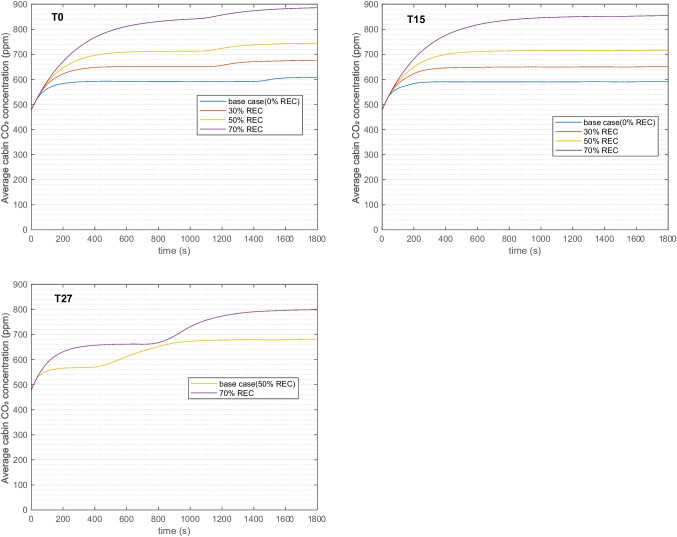
Cabin CO_2_ concentration profile at three ambient conditions, grouped with different recirculation degrees, in comparison with base case. One passenger is simulated

As shown in Fig. 7, the average cabin CO_2_ concentration increases when the recirculation is increased. The CO_2_ concentrations did not reach 1000 ppm in any case due to only 1 passenger is simulated. Although 70% REC increased this level to above 800 ppm for all climates.

The general elevating trend exhibit slight difference at three temperatures, as a consequence of the climate control, which adjusts the fan rpm at different stages of the cycle. For T27 case, the initial fan rpm is slightly higher than the other two (around 3000 rpm compared with around 2000 rpm); later, the fan rpm is decreased to a similar level for all three temperatures, which explains the more apparent increase of CO_2_ around 800 s for T27.

#### Energy consumption

**Fig. 8:**
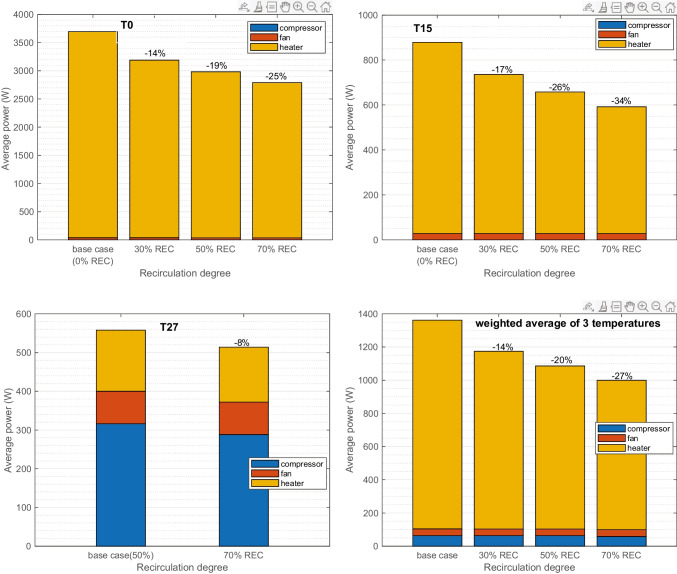
Case average power of compressor, heater, and fan at T0, T15, and T27. Weighted average of the three conditions is calculated (weight 0.2, 0.6, and 0.2). Results are compared at different recirculation degrees

Figure 8 presents the climate power case average (30 min) when REC is increased. The results are showing descending trends at all three climates. At T0, the outside air is heated up before entering the cabin. Increased recirculation elevates the temperature of mixed incoming air to the HVAC, which results in less heating demand. The applied strategy is similar in T15 in this study (AC off), and thus, similarly less heating power is consumed. At T27, when the cooler cabin air is mixed with warmer outside air, the dominant compressor power required is reduced.

When 70% REC applied, the total power reduced by 25%, 34%, and 8% respectively for T0, T15, and T27. Overall weighted average total power is reduced from 1.4 to 1.0 kW at 70% REC, i.e., 27% reduction.

### Increased recirculation with 2 to 5 passengers: influence on CO_2_ concentration and fogging

In the previous simulations, increased REC shows advantage in reducing climate power and particle concentrations in all three climates. The elevation on CO_2_ concentration is also limited. However, in those simulations, only the common scenario of 1 passenger is considered. The main climate power and particle concentration are generally not influenced by passenger numbers in the used model, but the CO_2_ concentration could be largely influenced due to passenger respiration. To further understand this influence, simulations were performed for increased recirculation with number of passengers varying from 2 to 5. The results are still compared with the target level of 1000 ppm.

**Fig. 9:**
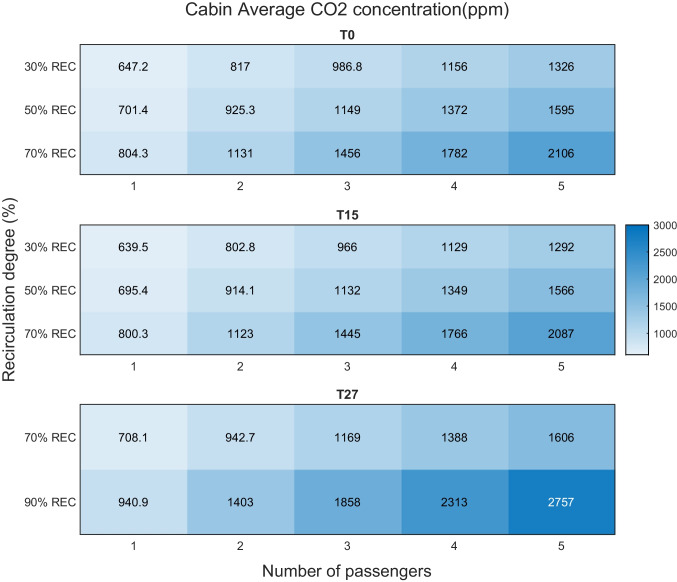
Heatmap of cabin CO_2_ concentration (30-min case average) for increased recirculation simulations. Number of passengers are varied from 1 to 5. Ambient condition T0, T15, and T27

A heatmap of the 30-min case average CO_2_ concentrations are shown in Fig. 9. All three temperature results are similar that up to 70% REC has an average result lower than 1000 ppm for 1 passenger. While as the passenger number increases, the acceptable REC degree is declining. For example, at T0 and T15, 30% REC is not recommended with more than 3 persons. When it comes to T27, the slightly higher ventilation airflow is beneficial in maintaining a lower CO_2_ concentration. Yet, 70% and 90% REC is not recommended when passengers are more than 2 and 1, respectively.

Besides, the windscreen fog risk is predicted. The average windscreen temperature and the average cabin air dew point temperature are compared continuously in the 30-min cycle. If the difference between the two is smaller than 5 °C, it is considered there might be a windscreen fog risk. The risk only exists in cold climate, not in intermediate and warm climate. Table 5 shows the length of time that fog risk is identified in the whole 30-min cycle, in the cold climate with different REC levels. It should be noted that the ventilation distribution in cold climate in this study is simplified as floor/defroster mode all through, which means more airflow is directed to the windscreen than in real conditions. It possibly reduced the occurrence of fogging in the simulation.

**Table 5:** Total time of potential fog risk in each simulation cycle in cold climate (T0)

Passenger numbers	30%REC	50%REC	70%REC
1	0 min	0 min	0 min
2	0 min	0 min	0 min
3	0 min	0 min	0 min
4	4 min	6 min	21 min
5	7 min	12 min	27 min

The results indicate that application of high REC in cold climate should be critically evaluated to avoid fog risks. It is a balance between energy saving and risk avoidance. Windscreen fogging is mainly defined by windscreen temperature and cabin air dew point, which would be influenced by several factors where REC is only one of many. Increased use of cabin air temperature and humidity sensors in modern vehicles provides valuable inputs to the climate control. When the sensors are located at a proper position, the cabin air adjacent to windscreen could be measured with quick response. Another trend is to also measure the exterior air humidity and proactively adjust the climate system operation. The information of passenger number could improve the control performance. The control of air distribution (defroster flow) and AC are important output from climate control.

### Feedback control of recirculation degree based on CO_2_ concentration

The above investigation showed that the increased recirculation is beneficial in reducing particle concentrations in all climates, and in improving energy efficiency in cold, warm climates and in intermediate climate when AC is off (the strategy in this study). In cold climate, the potential windscreen fog risks exist, which means increased REC requires more critical judgement. In all climates, the accumulation of CO_2_ highly depends on the passengers and ventilation airflows. Benefiting from a higher ventilation airflow in the studied T27 cases, higher REC is more likely to implement. Thus, T27 is now further studied in the feedback control sample on REC.

The feedback is based on the cabin CO_2_ concentration which is not higher than 1000 ppm. The simulation is based on the ambient condition T27, medium outside particle concentration level, an aged filter, and 1, 2, 3, 4, and 5 passengers separately.

**Fig. 10:**
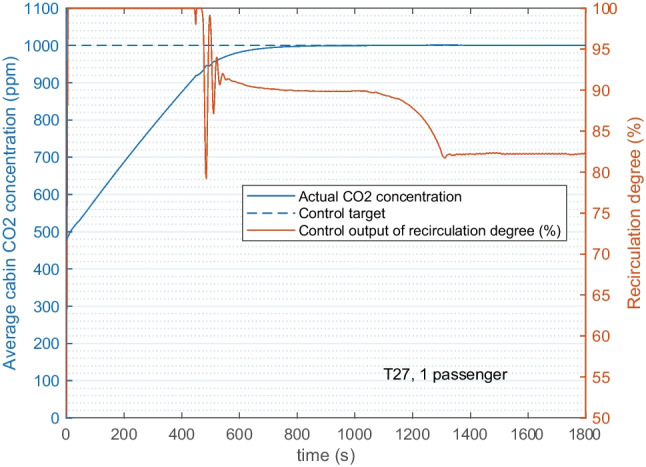
Feedback control of recirculation degree based on the cabin CO_2_ concentration. The control target is not higher than 1000 ppm

**Fig. 11:**
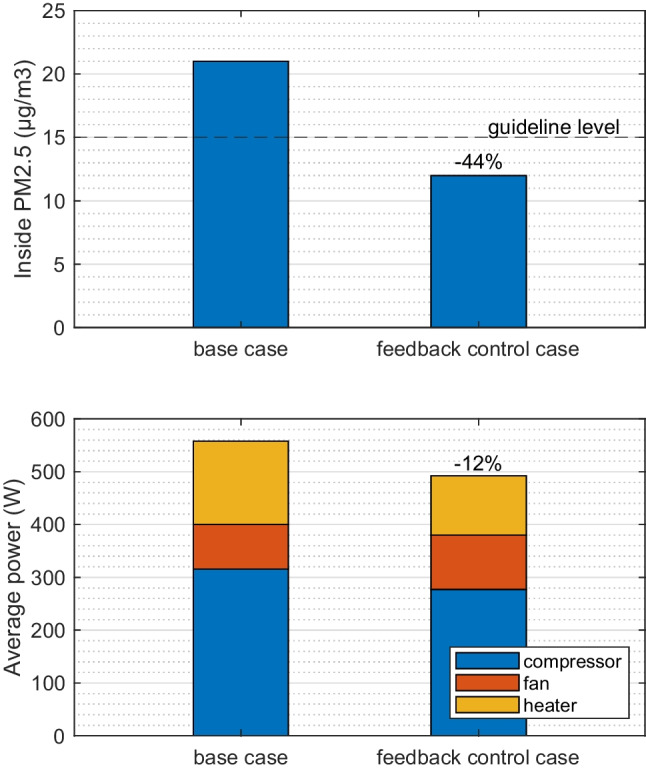
Comparison of base case and feedback control case results of steady state PM_2.5_ concentration and case-averaged power. Ambient condition T27, medium outside particle concentration level, aged filter, and 1 passenger

Figure 10 shows the control results for 1 passenger. The REC starts with 100%, then as CO_2_ accumulates the REC dips down and finally stabilizes, which leads to a 30-min average of 90% REC. The CO_2_ concentration is not higher than 1000 ppm throughout the whole 30-min simulation. At the same time, the increased REC from base case (50%) to 90% introduced a reduction on the particle concentration and climate power. As shown in Fig. 11, the steady state inside PM_2.5_ and case average of the total power were reduced by 44% and 12% correspondingly. Even with an aged filter adopted, the inside PM_2.5_ was reduced to 12 μg/m^3^ which is lower than the guideline level of 15 μg/m^3^.

**Fig. 12:**
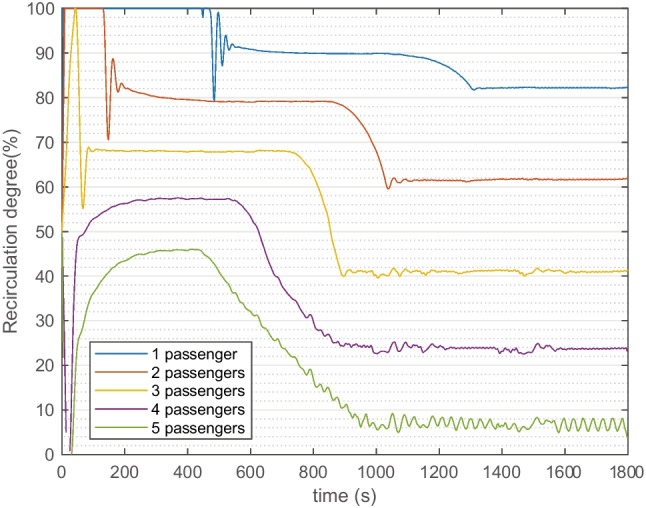
Controlled output of recirculation degrees (%) in the feedback control simulation. Passenger numbers from 1 to 5 are compared. Ambient condition T27

**Fig. 13:**
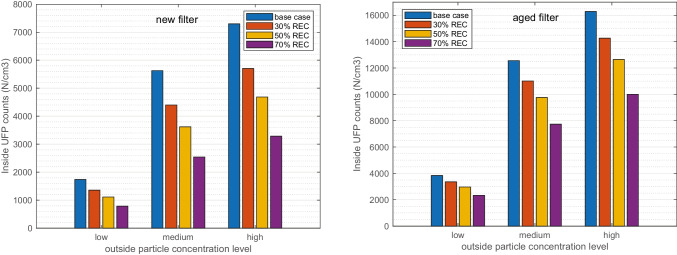
Steady state inside UFP counts at three outside concentration levels, grouped with different recirculation degrees, in comparison with base case. Low, medium, and high stand for outside PM_2.5_ concentration, which are 18, 48, and 128 μg/m^3^ correspondingly. Each bar is the weighted average of cold

The simulation was also performed on 2 to 5 passengers with the same configuration. The controlled CO_2_ concentrations are similar while the results of different REC are shown in Fig. 12. The REC decreases when more passengers are in the cabin. When there are 2, 3, 4, and 5 persons, the corresponding controlled REC (30 min-average) are lowered to 74%, 54%, 34%, and 18%, respectively.

## Discussion

The simulated cases and control strategies are designed to be representative of the real vehicle running conditions. There are however some things that have an influence on the simulation results to some extent. Concerning outside particle concentration and distributions, three profiles from real road testing were selected according to the known air quality level guidelines (European Environment Agency 2013). Yet, the particle distribution may have variation in different locations and road conditions (Zhu et al. 2007; Qiu et al. 2019), so using other particle data will influence the result. The climate control strategies adopted might also influence the results. In the simulated scenarios, a relatively lower fan rpm adopted leads to a higher accumulation of CO_2_ in relation to real conditions.

There are also some necessary simplifications in the simulation, which in one way leads to an effective study, while on the other hand, might have an influence on the results. For example, as mentioned that the air distribution in 0 °C condition is constant floor/defroster mode in the whole 30-min cycle. In reality, the airflow to defroster is lessened as the cabin is heated up. This means more airflow is directed to the windscreen in the simulation, which potentially induces lower fog risks and slightly slower heating up of the whole cabin in comparison with real conditions.

Another simplification is that the recirculation is set at a fixed level in most of the cases. While in reality, it is controlled by several factors including the exterior air quality sensor, the air temperature, and humidity in the cabin, etc., to maintain a good air quality level and climate performance. This may have influence on the results. For example, the REC could be elevated temporarily when the exterior pollution is detected high. This would probably cause more accumulation of CO_2_ in real conditions.

The guideline value of 1 000 ppm for CO_2_ could probably be higher for shorter periods. It is a common value used to control ventilation in occupied spaces in buildings, and it is a level that people can notice entering a room. A higher value would give less restrictions on the number of passengers.

The feedback control example presented investigates the possible control of ventilation settings, in this case the REC, to maintain a desirable CO_2_ level in the cabin. It could also be extended to wider applications, for example, controlling the fog risk in cold climate simultaneously. Moreover, in future studies, the control strategy could be enhanced and combined with existing control of HVAC fan based on the cabin air temperature and humidity. The input parameters could include cabin CO_2_ sensor, outside/inside temperature and humidity sensors, outside air quality sensor, and passenger number to maintain the good air quality and climate comfort level.

The control strategy could also be enhanced in the measures adopted. In addition to reducing the REC to decrease CO_2_ concentration, it might also be an effective way to temporarily increase the ventilation airflow, which would have different influence on the total energy consumption. These strategies could be evaluated in a future study.

## Conclusion

The strategy of increased air REC in vehicle climate system is investigated with simulations for three ambient conditions (cold, intermediate, and warm) and three outside particle concentration levels (low, medium, and high). The focus is REC influence on cabin air quality (particles and CO_2_) and the energy (power) required to maintain the desired cabin air temperature under average driving conditions. Few simplifications on the climate control strategy exist compared with production vehicle. The conclusions are for a specific vehicle (Volvo XC90 model-year 2018) and specific simulation conditions. However the tendencies are likely universal considering the study setup.

Overall increased REC is beneficial in reducing particle concentrations in all climates, and in improving energy efficiency in cold, warm climates and in intermediate climate when AC is off (the strategy in this study). REC level is limited by CO_2_ accumulation. In warm climate there is less restriction of CO_2_ due to a higher fan speed applied in the studied system. In cold climate REC is also limited by the fogging risk. Passenger number has a major influence on these limitations.

Originally in the studied vehicle when the new filter is installed, WHO PM_2.5_ guideline level of 15 μg/m^3^ (WHO 2021) could be reached in most cases. The aged filter has reduced performance. When one passenger is seated, the CO_2_ concentration is always below 700 ppm. The climate power is the highest at 3.7 kW in cold climate due to the heating demand, and lowest in warm climate at 0.6 kW where the main contribution is the compressor. The weighted average of all climates is 1.4 kW.

Increasing REC to constant levels reduces the particles similarly in all three climates. The application of 30%, 50%, and 70% REC on average reduced the PM_2.5_ concentration by 22%, 36%, and 55%, respectively, with a new filter. For an aged filter, the corresponding reductions are slightly less. Regarding the required power, in cold and intermediate climates, the improvements are more obvious. The average power is reduced from 1.4 to 1 kW.

As REC raised to 70% the CO_2_ accumulates but always below the guideline of 1000 ppm with 1 passenger seated. When passengers increase, the CO_2_ concentration rises considerably. In the cold climate at 70% REC, it climbs from 804 ppm with 1 passenger to 2106 ppm with 5 passengers. The corresponding 70% REC concentration at T15 and T27 with 5 passengers are 2087 and 1606 ppm. Adopting the guideline level of 1000 ppm, the allowed REC levels for different passengers are concluded and providing insights to the climate control design. In warm climate, 70% REC is not recommended with more than 2 people and not more than 1 person in cold and intermediate climates. In cold climate, the potential windscreen fogging risk is enlarged with REC with more than 3 persons.

Apart from a constant REC level, a dynamic feedback control on REC in warm climate presents the application of maintaining CO_2_ concentration below 1000 ppm while maximizing the reduction of particles and climate power. When 1 passenger is simulated, the average REC is increased from 50 to 90%; meanwhile, the cabin PM_2.5_ reduced by 44% and climate power reduced by 12%. When there are 2, 3, 4, and 5 persons, the corresponding controlled average REC are lowered to 74%, 54%, 34%, and 18%, respectively. Future studies could combine the strategy with cabin and outside sensors to improve energy efficiencies, air quality, and maintain climate comfort, which for example has been investigated in building HVAC application and offered 50% energy saving (Che et al. 2019).

## Data Availability

The datasets used and/or analyzed during the current study are available from the corresponding author on reasonable request.

## Appendix

Tables

**Table 6:** Mass and count concentration of particles within 10 nm and 2530 nm, for the three ambient particle profiles

Particle size channels (nm)																									
	10	14	19	27	37	52	72	100	139	193	253	298	352	414	488	576	679	800	943	1112	1310	1545	1821	2146	2530
Mass concentration (μg/m^3^)																									
Low	0.0	0.0	0.0	0.0	0.1	0.4	1.0	1.3	0.7	0.5	0.8	0.7	0.5	0.4	0.7	0.6	0.5	0.5	0.5	0.6	0.8	1.0	1.5	1.8	3.2
Medium	0.0	0.0	0.0	0.1	0.3	1.0	3.2	8.3	13.1	7.9	1.3	1.2	1.2	1.1	1.1	0.9	0.6	0.6	0.7	0.8	1.1	1.2	0.9	0.7	1.0
High	0.0	0.0	0.0	0.1	0.3	1.3	4.8	8.9	18.4	21.6	10.3	10.2	9.5	9.0	6.8	5.3	2.5	1.5	1.4	1.2	1.6	2.0	2.6	3.5	5.5
Count concentration (N/cm^3^)																									
Low	41.7	137.1	354.8	832.0	1535.5	1882.3	1724.3	854.0	181.1	52.3	44.4	24.1	10.1	5.4	5.1	2.7	1.4	0.9	0.6	0.4	0.3	0.2	0.2	0.2	0.2
Medium	85.1	275.2	701.8	1748.8	3361.8	4720.6	5768.6	5560.4	3262.4	810.8	71.5	38.6	24.2	14.1	8.0	4.1	1.6	1.0	0.7	0.5	0.4	0.3	0.1	0.1	0.1
High	208.6	744.3	1391.7	2668.3	4351.7	6361.7	8637.0	5974.3	4603.3	2221.7	561.4	338.2	192.9	112.4	51.9	24.7	7.1	2.6	1.5	0.8	0.6	0.5	0.4	0.3	0.3

^6^,

**Table 7:** Explanation of the European Air Quality Index levels based on PM_2.5_ concentrations. The index level is also based on other pollutants including NO_2_, O_3_, SO_2_, and PM_10_; in this table, only the relevant legends on PM_2.5_ are given

	Good	Fair	Moderate	Poor	Very poor	Extremely poor
PM_2.5_ concentration (μg/m^3^)	0–10	10–20	20–25	25–50	50–75	75–800

^7^,

**Table 8:** Size-dependent filter efficiency applied in the simulation. The values are given for different filter status (new and aged)

Particle size channel (nm)																									
Filter status	10	14	19	27	37	52	72	100	139	193	253	298	352	414	488	576	679	800	943	1112	1310	1545	1821	2146	2530
New	0.72	0.72	0.72	0.72	0.72	0.72	0.72	0.71	0.70	0.68	0.65	0.66	0.69	0.88	0.88	0.88	0.91	0.92	0.92	0.94	0.94	0.96	0.96	0.97	0.97
500 h-aged	0.40	0.40	0.40	0.40	0.40	0.40	0.37	0.33	0.33	0.29	0.29	0.27	0.27	0.52	0.52	0.52	0.55	0.56	0.56	0.58	0.58	0.60	0.60	0.61	0.61

^8^

Figure 13

## Declarations

### Disclaimer

The funder is not involved in the design, data collection and analysis, and manuscript writing of this study.
